# Challenges of analytical methods for the characterization of microsamples from David Alfaro Siqueiros mural painting

**DOI:** 10.1007/s00216-024-05633-x

**Published:** 2024-11-13

**Authors:** Adrián Mejía-González, Yareli Jáidar, Pablo Aguilar-Rodríguez, Sandra Zetina, Nuria Esturau-Escofet

**Affiliations:** 1 Instituto de Química, Universidad Nacional Autónoma de México, 04510 Mexico City, Mexico; 2 Instituto de Investigaciones Estéticas, Universidad Nacional Autónoma de México, 04510 Mexico City, Mexico

**Keywords:** PVAc resin, Nitrocellulose lacquers, Acrylic paints, NMR, FTIR, SEM–EDS

## Abstract

This study presents the analysis of seven microsamples from *Untitled Mural 3*, painted by David Alfaro Siqueiros between 1964 and 1972. The mural has never been restored, therefore preserving the materials used by the artist. The microsamples were analyzed using a combination of techniques: optical microscopy (OM), scanning electron microscopy with energy-dispersive spectroscopy (SEM–EDS), attenuated total reflection Fourier transform infrared spectroscopy (ATR-FTIR), micro-Fourier transform infrared spectroscopy (micro-FTIR) with reflection mode, and high-resolution nuclear magnetic resonance (NMR) spectroscopy. In this work, compounds common to those identified in the analysis of the contemporary mural *Trazos de composición piramidal* composition were identified, such as acrylic and nitrocellulose binders, titanium white and iron red oxide as pigments, and DEHP as a plasticizer. Unexpectedly, polyvinyl acetate (PVAc) and different nitrocellulose monomers were identified, in addition to chrome yellow instead of ochre, and a predominance of quartz, asbestos, and talc as fillers, instead of carbonates. The study confirmed that Siqueiros used multiple binders in successive layers in no apparent order, providing valuable insights into his artistic techniques. The research also proved an efficient methodology for studying very small and complex samples.

**Supplementary Information:**

The online version contains supplementary material available at 10.1007/s00216-024-05633-x.

## Introduction

Siqueiros is known for his innovative experimentation with modern materials and synthetic industrial paintings. Some previous studies have characterized his early use of nitrocellulose lacquers through 1935–1949 [1], but he kept experimenting with materials as acrylics and vinylics, and their use for outdoor painting, specially, in the last period of his life between 1960 and 1974, the period studied in this research project.

Between 1964 and 1971, Siqueiros painted several murals in his workshop, *La Tallera*, in Cuernavaca, Morelos. These panels were pioneering experiments with materials for outdoor monumental painting, studies for his most ambitious project: the *Polyforum* [2].

The focus of this study is *Untitled Mural 3*, one of four mural compositions that were exhibited outside *La Tallera*. The murals were done to test the durability of painting materials in outdoor environmental conditions. The *Untitled Mural 3* measures 9.60 by 19.79 m, it is composed of asbestos cement panels mounted in steel frames. The design is characterized by intersecting lines that form geometric shapes, predominantly triangles. While the mural primarily exhibits a white background, it incorporates black, ocher, red, and gray lines, and planes.

Between 2010 and 2012, *La Tallera* underwent an architectural transformation, becoming an exhibition space for contemporary art. The project involved the restoration and relocation of two of the four murals on the façade. The remaining two murals were dismantled and stored at Centro Nacional de Conservación y Registro del Patrimonio Artístico Mueble (CENCROPAM) warehouses.

In 2021, one of the murals that had been restored and relocated outside of *La Tallera* was subjected to a detailed study to gain an understanding of its painting technique. A key contribution of the aforementioned study was the identification, through microscopic and spectroscopic techniques, of acrylics within the earliest and intermediate layers of the samples corresponding to different formulas (possibly Politec® and Carboline® acrylics) as described in a periodical from that era.^1^ Unexpected lacquers of nitrocellulose were characterized in the surface layers, with the presence of secondary resins and plasticizers identified as phthalates. The findings of the study prompted further inquiries regarding the authenticity of the lacquer layers, particularly in the context of whether they originated from the artist’s final campaign or were introduced during restoration treatments [3].

For this reason, the aim of this study was the characterization of *Untitled Mural 3*, which has no conservation treatment.^2^

Due to the complexity of the materials and the limited amount of sample in heritage analysis, it is of great importance to carry out studies using complementary analytical techniques. In particular, the analysis through a set of microscopic and spectroscopic techniques has proven to be very useful for the identification of the painting materials. The optical microscopy (OM) allowed the observation of the painting stratigraphy. Scanning electron microscopy with energy-dispersive spectroscopy (SEM–EDS) was employed to investigate the microstructure and to ascertain the inorganic elemental composition of particles. Attenuated total reflection Fourier transform infrared spectroscopy (ATR-FTIR) was utilized to identify inorganic and organic compounds, in a manner analogous to micro-FTIR, which permitted the identification of the painting stratigraphy. Finally, high-resolution nuclear magnetic resonance (NMR) spectroscopy was applied to elucidate organic compounds in these complex painting mixtures [3–7].

This research proposes the study of microsamples taken from *Untitled Mural 3*, as an opportunity to confirm that Siqueiros used nitrocellulose lacquers alongside acrylics between 1964 and 1972. The study also deepens our understanding of the artwork, the artist’s techniques, and the historical context in which it was created, while also providing valuable information for its conservation and preservation.

## Materials and methods

**Fig. 1:**
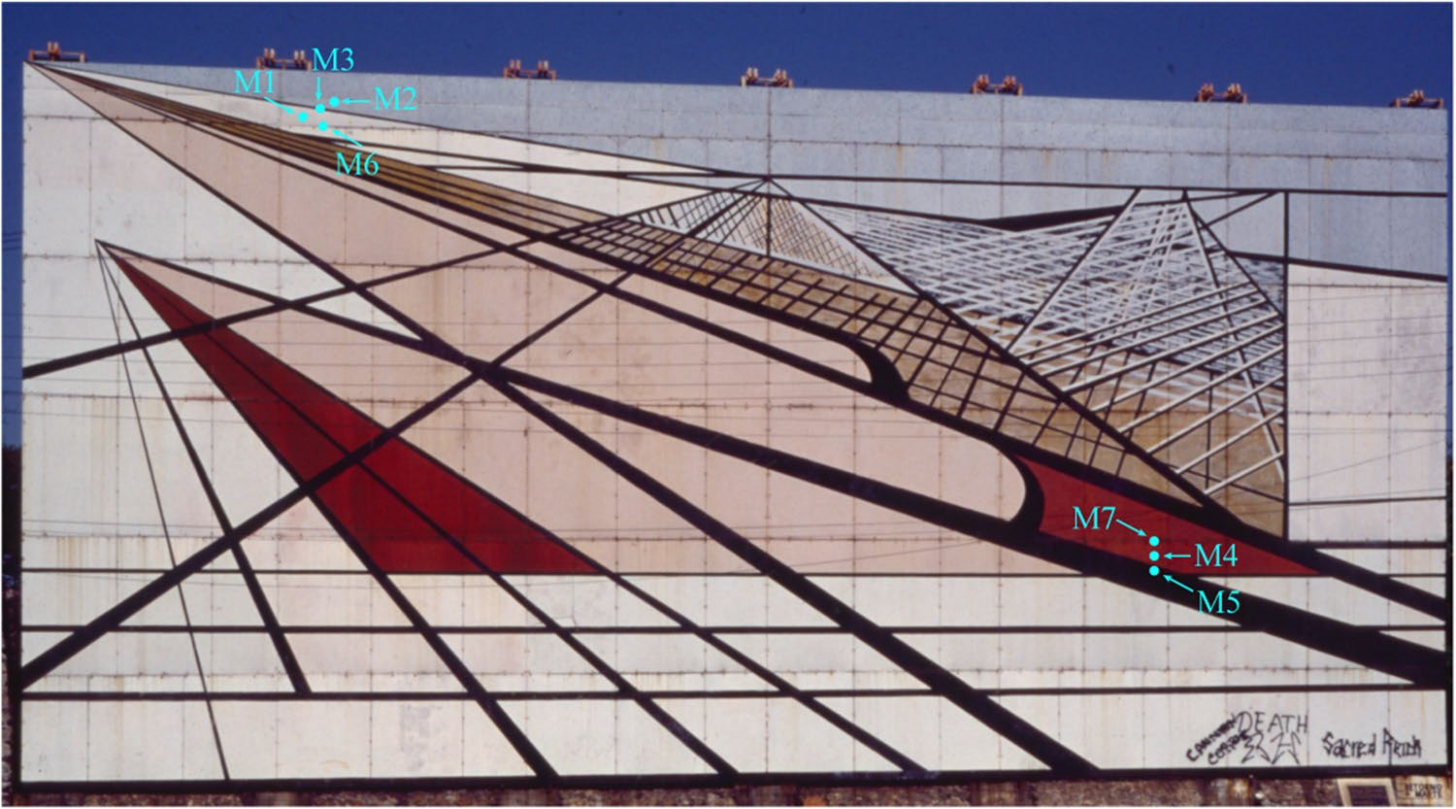
David Alfaro Siqueiros, *Untitled Mural 3*, ca. 1990s Photo: Ernesto Peñaloza, AFMT IIE UNAM. Sample locations are indicated

The seven samples were obtained by members of the *Laboratorio Nacional de Ciencias para la Investigación y Conservación del Patrimonio Cultural* (LANCIC), who had access to two of the panels stored by CECROPAM. *Untitled Mural 3* measures 9.60 m high by 19.79 m long and consists of 36 panels of various sizes: 24 sections of 1.83 × 3.30 m, 6 sections of 0.73 × 3.30 m, and 6 more sections of 1.50 × 3.30 m (Fig. 1). The microsample dimensions range between 0.5 and 1 cm^2^.

For microscopic analysis, a fragment of each sample was prepared as cross-section in a commercial unsaturated polyester resin (80–100%) and styrene (10–20%) Struers Clarocit®. The samples embedded in the resin were polished until the original material was exposed while the process was monitored through an OM. For ATR-FTIR, the front and back sides of the sample were analyzed directly. Meanwhile, for NMR analysis, another microscopic fragment was solubilized.

### Surface and optical microscopy

The surface of the sample was observed with an optical fiber microscope VHX-2000 SM (Keyence, Japan) and the micrographs were taken with 1600 × 1200 pixels resolutions at 20 × , 50 × , and 100 × . The cross-section of the sample was studied with an Axio Imager Z2 optical microscope (Carl Zeiss, Oberkochen, Germany) equipped with a Xenon arc lamp for UV fluorescence with 430–465 nm and 465–500 nm filters and a HAL100 light source in reflected light mode.

#### SEM–EDS

The electron micrographs of the sample cross-section were acquired with a Scanning Electron Microscope EVOMA25 SEM (Carl Zeiss, Oberkochen, Germany) primarily with a backscattered electron detector. An accelerating voltage of 20.0 keV was applied using a variable pressure of 80 Pa under a nitrogen flow to prevent the formation of electrostatic charges on the samples’ surfaces. The chemical elemental analysis of the samples was conducted using an energy-dispersive spectroscopy microprobe with a 30 mm diameter (Bruker, Germany). The sample was held in place and rendered conductive using double-stick carbon tape.

#### ATR-FTIR

ATR-FTIR analyses were performed with the Cary 600 spectrophotometer (Agilent Technologies, USA) in Total Attenuated Reflectance (ATR) mode with a diamond crystal. The spectra were acquired with 128 scans and a spectral range between 4000 and 400 cm^−1^ with 4 cm^−1^ resolution. Data was processed with Origin and Spectragryph v. 1.2 software. The identification of the signals was compared with literature and with IRUG spectral database [8, 9].

### Micro-FTIR

Micro-FTIR analyses were performed on cross-sections with an Agilent Cary 620 FTIR microscope (Agilent Technologies, Santa Clara, CA, USA) with a 64 × 64 Focal Plane Array (FPA) detector cooled by liquid nitrogen, coupled to an Agilent Cary 660 FTIR spectrometer. Data were collected and processed using Agilent Resolutions Pro software (Agilent Technologies, Santa Clara, CA, USA). The Field of View (FOV) was 70 × 70 μm. Spectra were acquired at 1.1 μm per pixel resolution, in the spectral range between 4000 and 900 cm^−1^, performing 1000 scans at 4 cm^−1^ resolution.

#### NMR

NMR spectra were acquired with a Bruker Avance III HD 700 spectrometer (Bruker, Billerica, MA, USA) operating at 16.4 T (700 and 175 MHz for ^1^H and ^13^C frequency, respectively) equipped with a 5-mm z-axis gradient TCI cryoprobe. The sample was dissolved in 0.6 mL of CDCl_3_; subsequently, the solvent was volatized at room temperature and then dissolved in DMSO-d_6_. The ^1^H-NMR and 2D-NMR experiments: edited heteronuclear single quantum correlation spectroscopy (edited-HSQC) and heteronuclear multiple-bond correlation spectroscopy (HMBC), were acquired at 300 K with the standard pulse sequences from the Bruker library and processed with MestReNova software (v. 14.0, Mestrelab Research SL, Santiago de Compostela, Spain). The chemical shifts (δ) are reported in ppm relative to the solvent resonance (CDCl_3_: δ^1^H = 7.26 and DMSO-d_6_ = 2.50 ppm).

## Results and discussion

### Optical microscopy and SEM–EDS

**Fig. 2:**
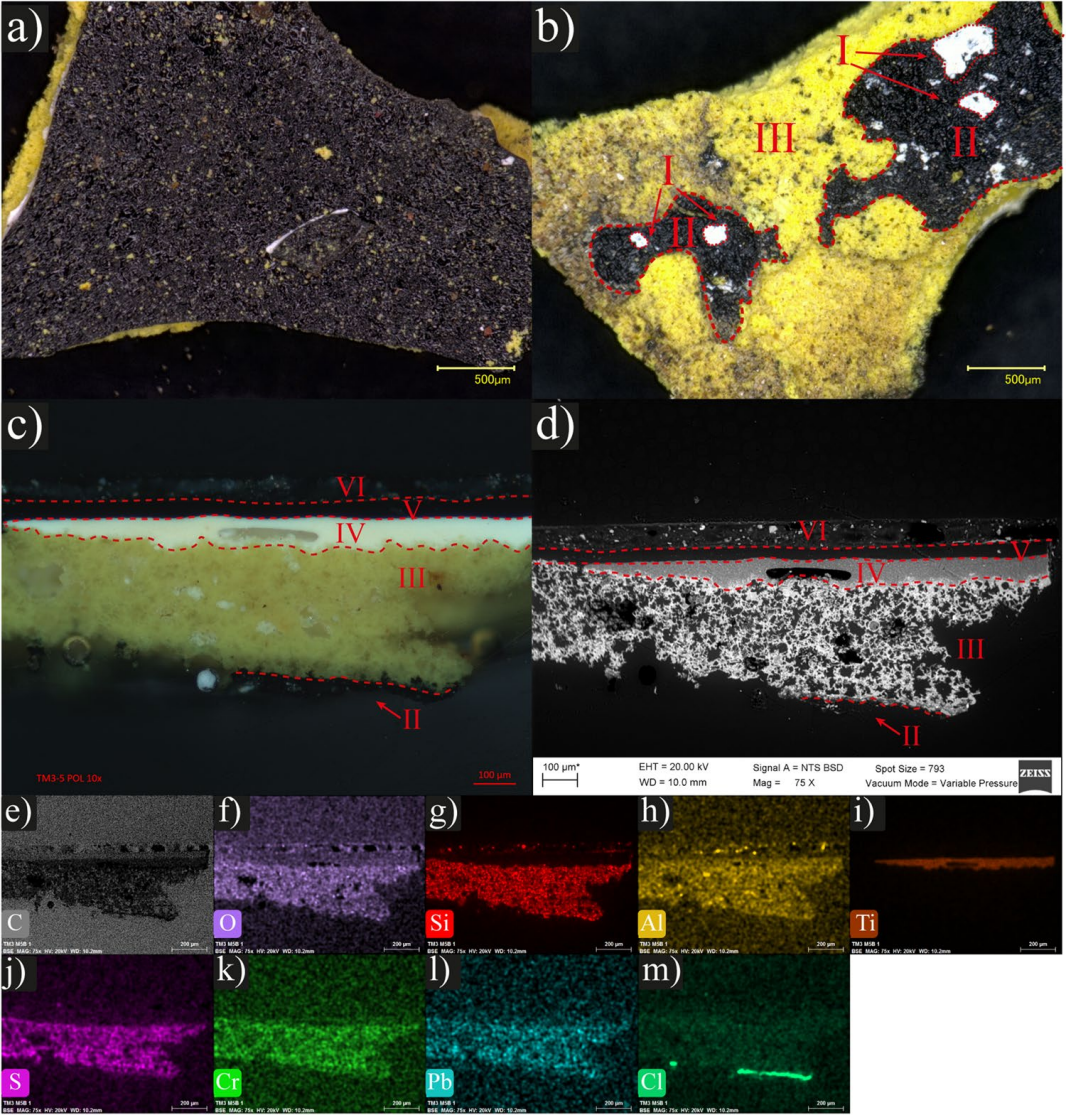
Micrographs and elemental mappings of the mural sample **5**. Optical micrographs: **a** frontal layer black **5-Vl** (20 ×), **b** back of the sample with residues of layers: white **5-I**, black **5-II** and yellow **5-III** (20 ×), and **c** stratigraphic cross-Sect. (5 ×). **d** SEM micrograph (BSE, 20.0 kV, 75 ×) of the cross-section. Elemental mappings (EDS, 20.0 kV, 75 ×) of predominant elements: **e** C, **f** O, **g** Si, **h** Al, **i** Ti, **j** S, **k** Cr, **l** Pb, and **m** Cl

Through OM, the samples were found to consist of a complex multilayered system, with each layer displaying distinct characteristics, color, composition, and thickness. Furthermore, the SEM–EDS analysis enabled the observation of the micromorphology of the inorganic components, along with the identification of the respective elemental composition (Table S1). In this way, the samples of the mural were analyzed, and as an example, the optical micrographs and the SEM–EDS analysis of sample **5** are presented (Fig. 2). The front black painting layer corresponds to the surface of the painting, which presents few detachments that reveal the underlying yellow layer (Fig. 2a). On the back, few residues of the first two painting layers were observed over the yellow layer: a barely notable white layer over a black layer. Despite the minimal amount of some pictorial layers available to be analyzed, it is important to consider the existence of the layers for the comprehensive analysis. Thus, the numbering of pictorial layers was assigned starting with the one closest to the mural panel: white **5-I**, black **5-II**, and yellow **5-III** (Fig. 2b). In the cross-section, it was observed the stratigraphy of the sample: departing from the black layer **5-II**, yellow **5-III**, white **5-IV,** black **5-V**, and black **5-VI** (Fig. 2c). The SEM micrograph of the same section illustrates the distinctive microstructures observed within each layer, particularly the extensive porosity of the yellow layer (Fig. 2d). The mapping of the elemental composition identified: C, O, Si, Al, Ti, S, Cr, Pb, and Cl (Fig. 2e–m). Based on elemental quantification, the predominance of C (50–60%) and O (25–37%) was related to the organic composition of the painting layers like the binders and additives which are the majority components of the formulations (Fig. 2e, f). The identification of Si (1.4–6.8%) was related to silicates used as fillers and traces of Al (0.1–0.3%). These fillers predominate in the yellow **5-III** and are dispersed in the black **5-V** and **5-VI** layers (Fig. 2g, h). In addition, Ti (0.3–8.4%) was identified with the highest concentration in the white layer **5-IV** and was related to titanium dioxide (TiO_2_), a common compound used as a white pigment. In the rest of the layers, it was also identified but less than 1% (Fig. 2i) [8]. S, Cr, and Pb were identified in the yellow **5-III** (Fig. 2j–l), which correspond to the inorganic pigment chrome yellow, a mixture of lead chromate with lead sulfate (PbCrO_4_ + PbSO_4_) [10, 11]. Cl was detected primarily in the black layer **5-II**, which may be associated with a component of the mural panel, such as an additive or a compound utilized in a previous fumigation (Fig. 2m). The elemental composition of each painting layer is presented in Table S2.

In the analysis of the pictorial layers of the other samples, it was observed that most of them contained a similar elemental composition. In some layers, it was also possible to identify particles composed of Na, Mg, K, Ca, and Fe, and in some cases, particular microstructures such as asbestos fibers were identified (Fig. S1), along with diatom micro skeletons and several amorphous fillers, which had been previously identified in the study of other Siqueiros paintings [12].

#### ATR-FTIR

**Fig. 3:**
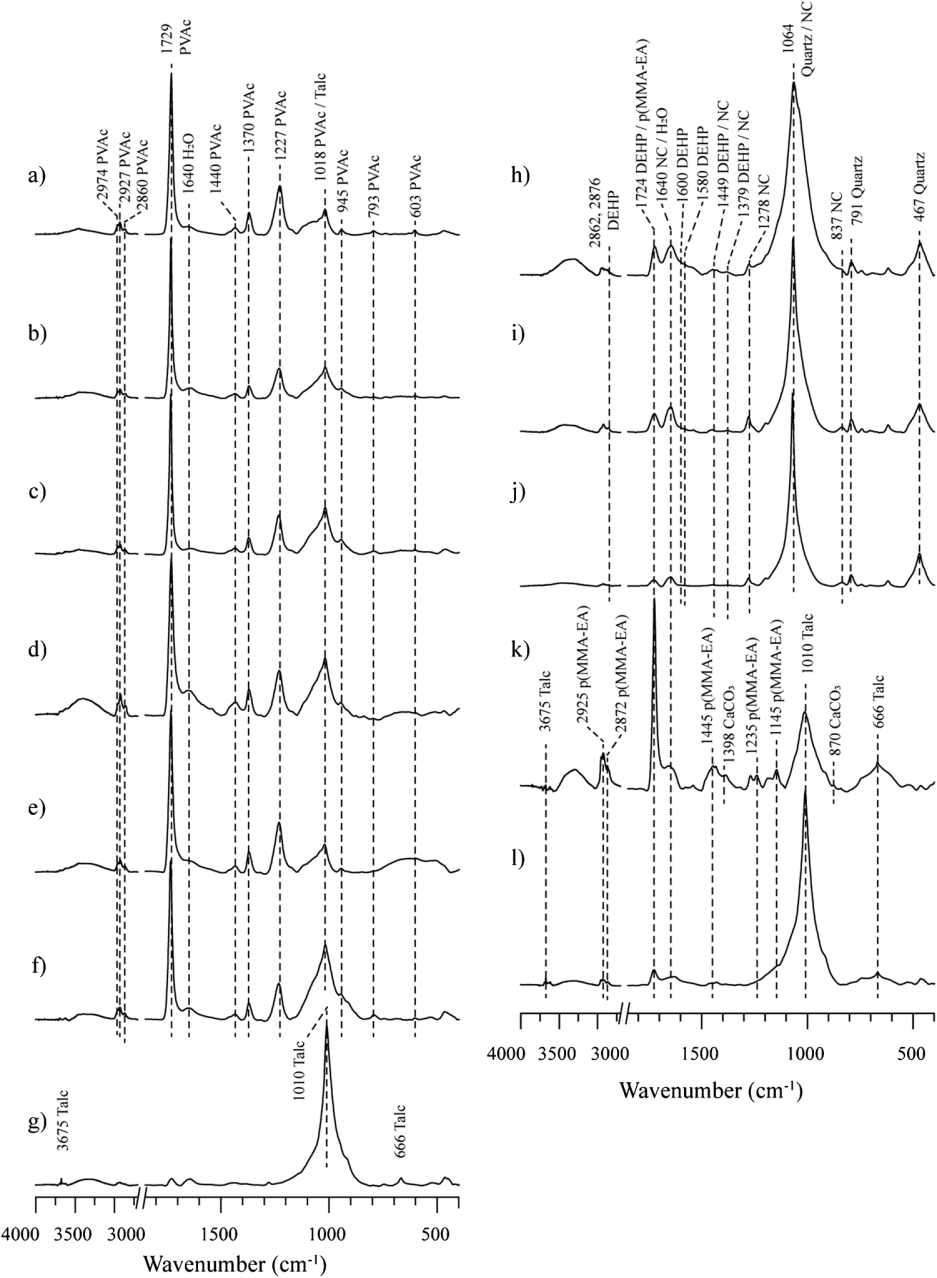
ATR-FTIR spectra from layers. Front: **a** black **5-VI**, **b** black **3-VI**, **c** white **1-IV**, **d** white **6-IV**, **e** beige **2-VIII**, **f** red **4-VII**, and **g** red **7-III**. Back: **h** black **5-II**, **i** yellow **4-V**, **j** yellow **7-II**, **k** white **1-I**, and **l** white **3-I**. Signals of PVAc, nitrocellulose, DEHP, EA-MMA acrylic binder, talc, quartz, and CaCO_3_ are indicated

Based on the binders and fillers identified in the analysis of the contemporary mural, the ATR-FTIR spectra of the materials were acquired to use them as reference proposals (Fig. S2). According to the analysis of sample **5**, the front and back layers of the other samples were studied (Fig. 3). The ATR-FTIR spectra of the black **5-VI** frontal layer and the black **5-II** back layers were acquired and analyzed directly. In the spectrum of the black **5-VI** frontal layer, the most prominent bands were identified and were found to be related to functional groups of polyvinyl acetate (PVAc) polymer resin. These included the following: 2974, 2927, 2860, 1729, 1440, 1370, 1227, 1018, 945, 793, and 603 cm^−1^, once these bands were assigned, the signal at 1640 cm^−1^ was observed along with the broad band at 3500–3200 cm^−1^ and were considered as the O–H bending and stretching, of water from ambient humidity (Fig. 3a). This composition was identified in all the frontal pictorial layers: black **3-VI** (Fig. 3b), white **1-IV** (Fig. 3c) and **6-IV** (Fig. 3d), beige **2-VIII** (Fig. 3e), and red **4-VII** (Fig. 3f). In the layer red **7-III** (Fig. 3g), signals were identified at 3675, 1010, and 666 cm^−1^ and were related to talc (Mg_3_Si_4_O_10_(OH)_2_).

Regarding the study of back painting layers, contrasting compositions were identified. In the spectrum of the black **5-II** back layer, absorption bands related to nitrocellulose were identified at 3500–3000, 1640, 1449, 1379, 1278, 1064, and 837 cm^−1^. Due to the OH group vibrations of water can overlap with the signals of nitrocellulose, in addition to the identification of characteristic signals such as N–O group, the relative intensity between the signals was considered. In addition, the bands of diethylhexyl phthalate (DEHP) were identified at 2876, 2862, 1724, 1600, 1580, 1449, 1379, and 743 cm^−1^. DEHP is a common plasticizer used in nitrocellulose lacquer formulations [13]. Additionally, the bands associated with silicates, possibly like quartz, due to characteristic bands at 1064, 791, and 467 cm^−1^, were identified (Fig. 3h). The same components were also identified in the spectrum from yellow back layer **4-V** (Fig. 3i) and in the spectrum from yellow **7-II**, where the DEHP and nitrocellulose signals are attenuated (Fig. 3j). On the other hand, in the spectra from white layer **1-I**, the bands identified at 2925–2872, 1724, 1445, 1235, and 1145 cm^−1^ were related to an ethyl acrylate-methyl methacrylate (EA/MMA) acrylic binder, along with the bands related to talc, and bands at 1398 and 870 cm^−1^ that were relate to calcium carbonate (CaCO_3_) since Ca was identified in the elemental analysis by EDS of this layer (Fig. 3k). The CaCO_3_ fillers were only identified in this pictorial layer. In the white layer **3-I** (Fig. 3l), bands related to talc and low intensity bands related to the acrylic binder were observed. Regarding the back side of samples **2** and **6**, ATR-FTIR spectra were not acquired since it had residues of support panel. The signals of the identified compounds are presented in Table 1.

**Table 1:** Identified compounds by ATR-FTIR in front and back painting layers

Sample layers	Color	Identified compounds						
		PVAc	Nitrocellulose	DEHP	Acrylic	Talc	Quartz	CaCO_3_
5-VI/F	Black	X						
3-VI/F		X						
1-IV/F	White	X						
6-IV/F		X						
2-VIII/F	Beige	X						
4-VII/F	Red	X						
7-III/F		X				X		
5-II/B	Black		X	X			X	
4-V/B	Yellow		X	X			X	
7-II/B			X	X			X	
1-I/B	White				X	X		X
3-I/B					X	X		

*F* front, *B* back

### Micro-FTIR

**Fig. 4:**
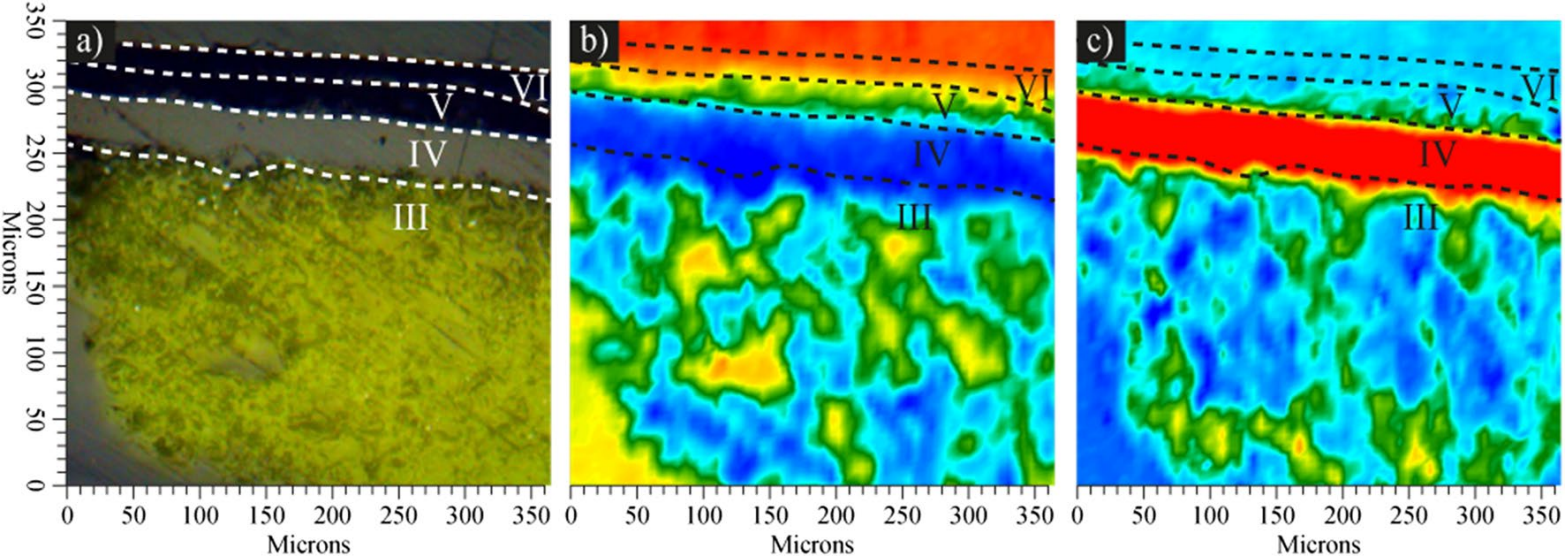
Micro-FTIR analysis from the mural sample. **a** Optical micrograph of the stratigraphic cross-section. Chemical mapping with the intensity distribution of the reflectance peaks of **b** signal at 1749 cm^−1^ related to the carbonyl group from acrylic and PVAc resins from black **5-V** and **5-VI** layers and **c** signal at 1678 cm^−1^ related to the nitro group from nitrocellulose in white **5-IV** and yellow **5-III** layers

Due to the stratigraphic complexity observed in the studied samples, the identification of the materials along the painting layers was complemented through micro-FTIR analysis. The analysis of the upper layers from sample **5** is presented with the respective chemical mappings (Fig. 4). The different layers of the stratigraphic cross-section were examined, and the results were correlated between the optical micrograph (Fig. 4a) and the 1.1 µm^2^ IR reflectance spectra, which form the chemical mapping. The chemical mappings of the surface were observed as areas of increasing intensity from blue to red (Fig. 4b, c). Based on the compounds identified in the ATR-FTIR spectra, the characteristic bands from the binders were related to the respective bands in the reflectance spectra from the chemical mappings and from characterized commercial samples. The analysis of the commercial samples was conducted to complement the general identification of PVAc (Fig. S3a) and nitrocellulose (Fig. S3b).

On the upper side of the optical micrograph from the microsample, a portion of the inclusion acrylic resin is observed (Fig. 4a). In the corresponding area of the chemical mapping, a signal was identified at 1749 cm^−1^ in the absorbance spectrum and was related to the stretching of the carbonyl group (C = O) from the acrylic resin. The signal was detected with a higher intensity (in red) and with less intensity (yellow and green areas) in the other painting layers, indicating that part of the sample is covered by the resin. At the same time, the band was identified and related to the PVAc resin from black layers **5-V** and **5-VI**, due to the chemical structural similarity and because the polymer was identified through ATR-FTIR spectroscopy (Fig. 4b). Conversely, in the region corresponding to the white layer **5-IV** and yellow **5-III**, a signal was identified with great intensity at 1678 cm^−1^. This could be related to the vibration of the nitro group (N–O), which would indicate the presence of nitrocellulose as a binder in these pictorial layers (Fig. 4c). Due to the discontinuity of the white **5-I** and black **5-II** layers, analysis by this technique was not possible.

The results of micro-FTIR and the identified binders in all the samples are summarized in Table S3.

#### NMR

Fractions of the multilayered microsamples were taken and solubilized in CDCl_3_ to be analyzed by NMR. In the ^1^H-NMR spectra, the corresponding compound signals of each mixture were observed, and through the 2D-NMR experiments, the structures of the major organic compounds were elucidated.

**Fig. 5:**
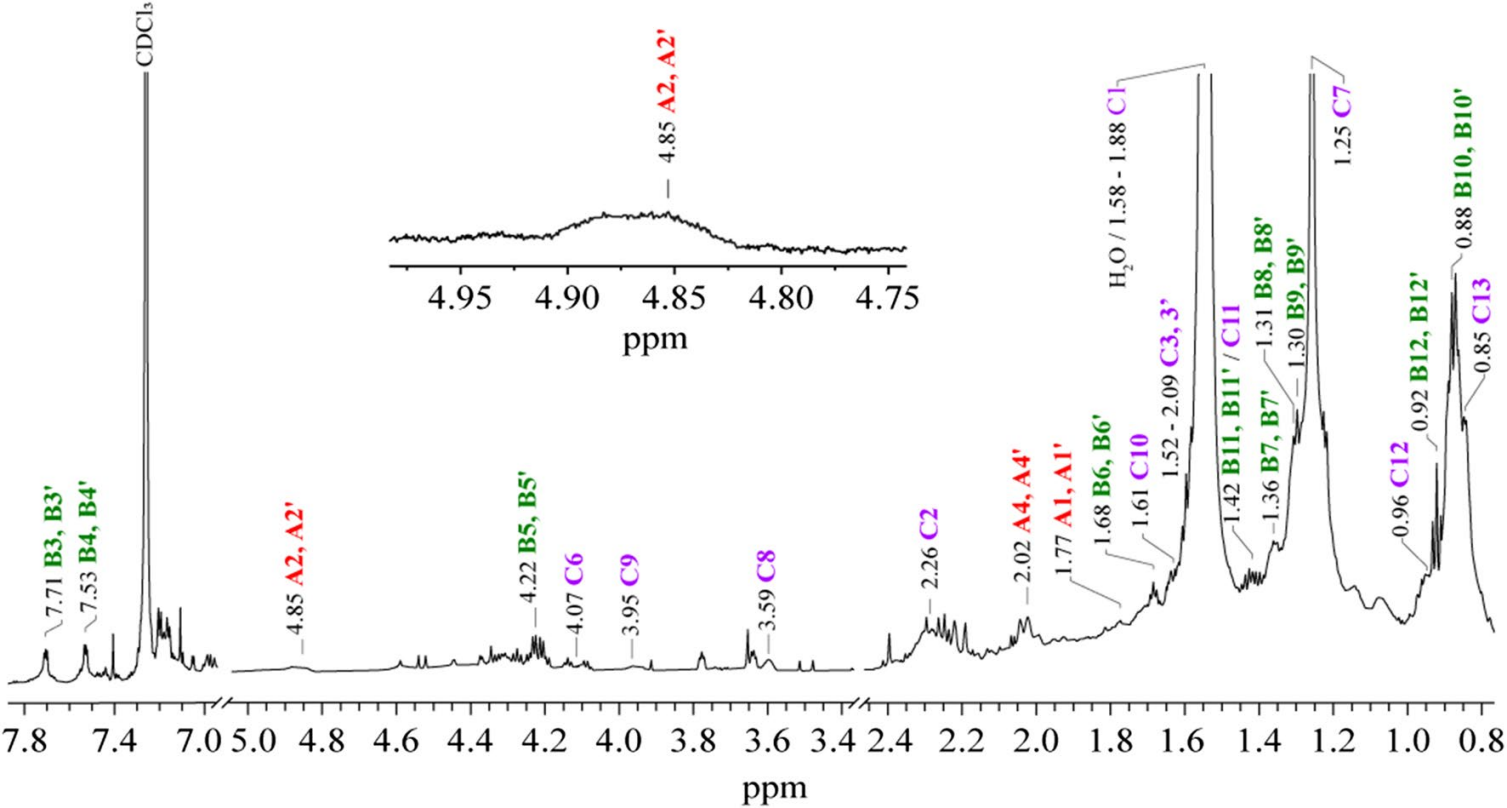
^1^H-NMR spectra (700 MHz, CDCl_3_, 300.0 K) of the mural sample **5**. The signals of PVAc, DEHP, EA, MMA, and nBMA are indicated

In the ^1^H-NMR spectrum in CDCl_3_ from sample **5**, several signals with different intensities were observed (Fig. 5). Some of these signals exhibited multiplicity and are narrow, indicating the presence of low-molecular-weight compounds, while broad signals were indicative of larger compounds. In the 2D-NMR spectra, the signals related to PVAc binder (Fig. S4a) and DEHP plasticizer (Fig. S4b) were confirmed since they were identified in the surface layers analysis through ATR-FTIR [14–16]. Additionally, the signals related to EA, MMA, and n-butyl methacrylate (nBMA) acrylic monomers were identified, which was not found in the superficial layers but was related to the intermediate layers of the sample. In the ^1^H-NMR spectra from the other samples, the signals of the same components were identified along the spectra with different intensities; in some cases, it was not possible to identify them (Fig. S4c). The chemical compounds and the ^1^H and ^13^C NMR data are listed in Table 2.

**Table 2:**
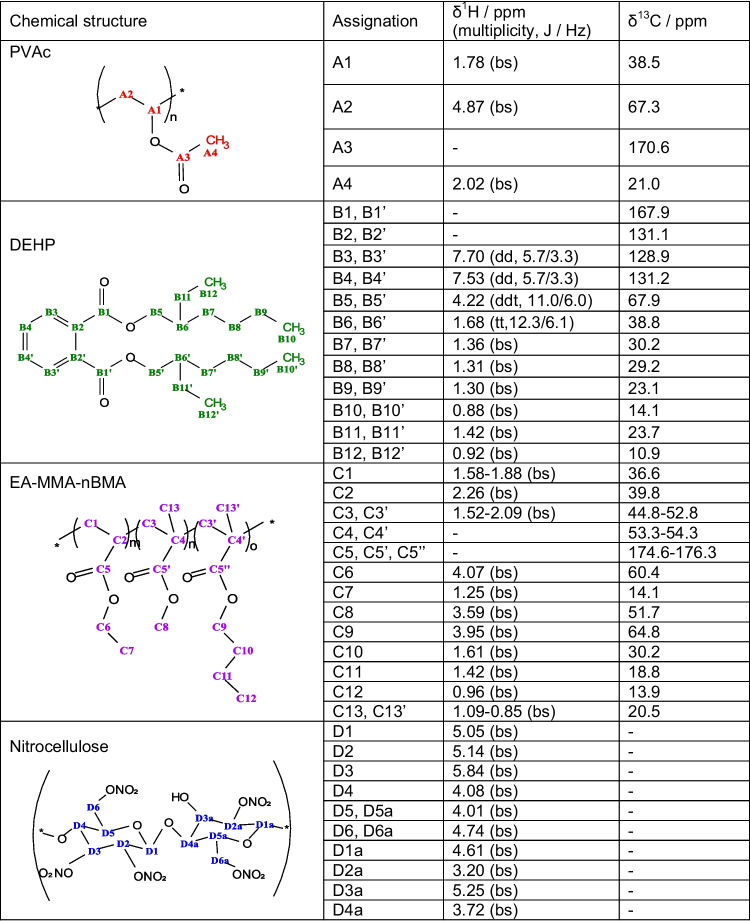
NMR data of the identified compounds in mural sample **5**

*bs* broad signal, *dd* doublet of doublets, *ddt* doublet of doublet of triplets, *tt* triplet of triplets

**Fig. 6:**
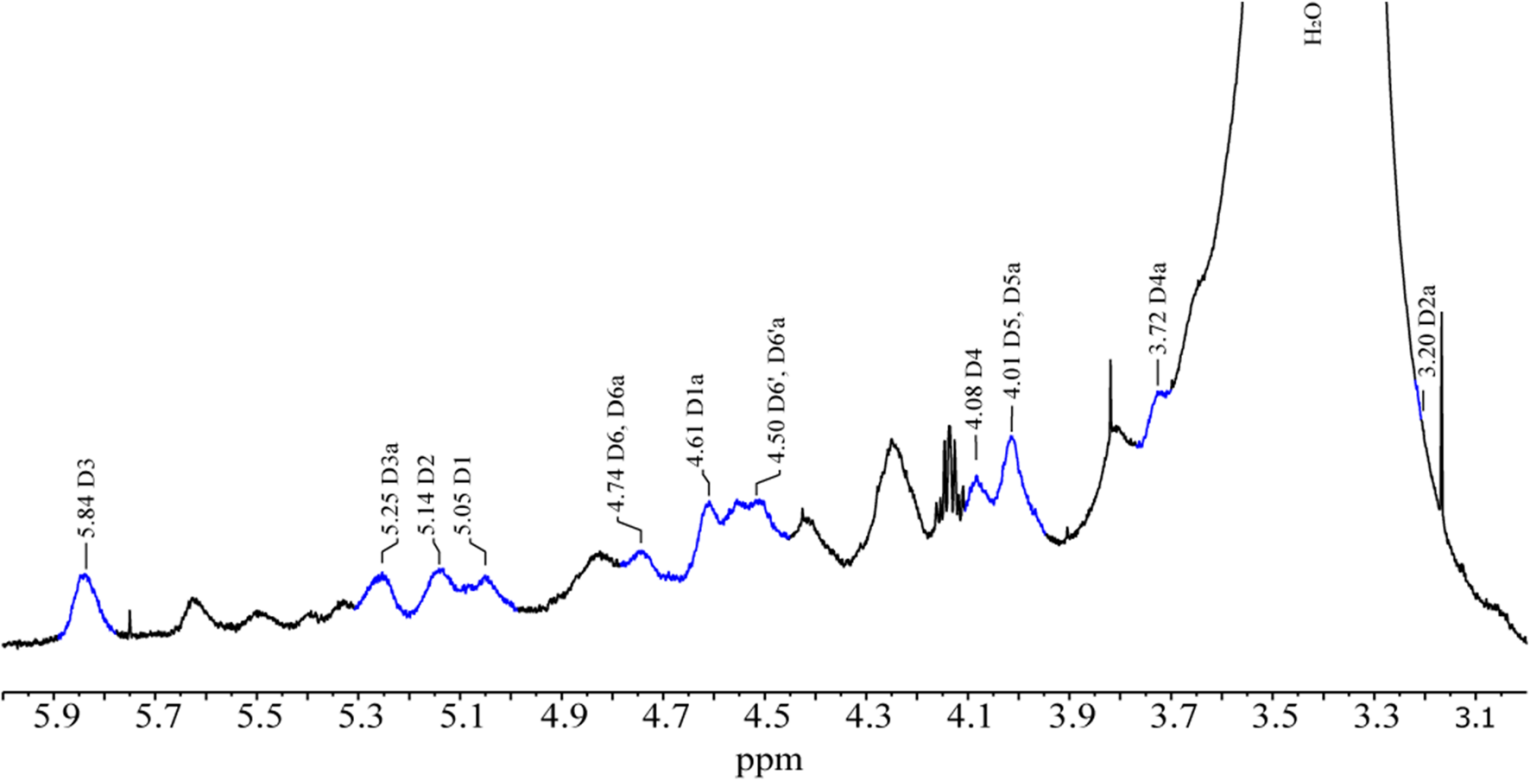
^1^H-NMR spectra (700 MHz, DMSO-d_6_, 300.0 K) of mural sample **5**. The signals of TNC and 3,6-DNC nitrocellulose monomers are indicated

Subsequently, the same sample was analyzed using DMSO-d_6_. ^1^H-NMR was focused on broad and low-intensity signals between 3 and 6 ppm that are related to nitrocellulose, based on the references where the characterization of the nitrocellulose monomers is addressed [17, 18]. Thus, in the ^1^H-NMR spectrum, the signals identified at δ^1^H = 5.05, 5.14, 5.84, 4.08, 4.01, 4.74, and 4.50 pm were related to the reported chemical shifts of the ^1^H from the assigned as D1–D6 from 2,3,6-trinitrocellulose (TNC) monomer, while the signals at δ^1^H = 4.61, 3.20, 5.25, 3.72, 4.01, 4.74, and 4.50 ppm were related to ^1^H from the assigned as D1a–D6a from 3,6-dinitrocellulose (3,6-DNC) monomer (Fig. 6). Additionally, a commercial sample of nitrocellulose lacquer was analyzed to confirm the proposed chemical composition. The sample was analyzed through ^1^H-NMR and edited-HSQC spectra, where the identified signals were related to two main monomers: TNC and 2,6-dinitrocellulose (2,6-DNC) (Fig. S4d). The ^1^H-NMR data of the identified nitrocellulose monomers in the mural sample are listed in Table 2. By proposing that the TNC monomer was present in both samples while the second monomer differs, it is possible to differentiate the 2 formulations used.

## Discussion

Table 3 provides a summary of the identification of organic and inorganic materials detected in seven samples of *Untitled Mural 3.* Firstly, the presence of silicates as inorganic fillers was associated with the ground or binder formulations, in very thick layers near to the support. Those layers were colored in yellow, white, beige, red, and black and were almost always associated with nitrocellulose lacquers. By the microstructure of the fillers and their composition, they were identified as asbestos particles. These findings are consistent with previously documented evidence that Siqueiros incorporated asbestos into his formulations, particularly into the ground layers, with the intention of modifying the fluidity of the painting and rendering nitrocellulose opaquer, thicker, and porous [12]. Additionally, in some layers, diatom micro skeletons, talc, and, on few occasions, CaCO_3_ were found. In the contemporary mural, *Trazos de composición piramidal*, previously analyzed, CaCO_3_ and MgCO_3_ aggregates predominated in most of the studied layers [3]. The comparison of the composition of the inorganic fillers and its association with the binders in both murals indicate that the formulations come from different brands or were prepared in dissimilar ways.

**Table 3:**
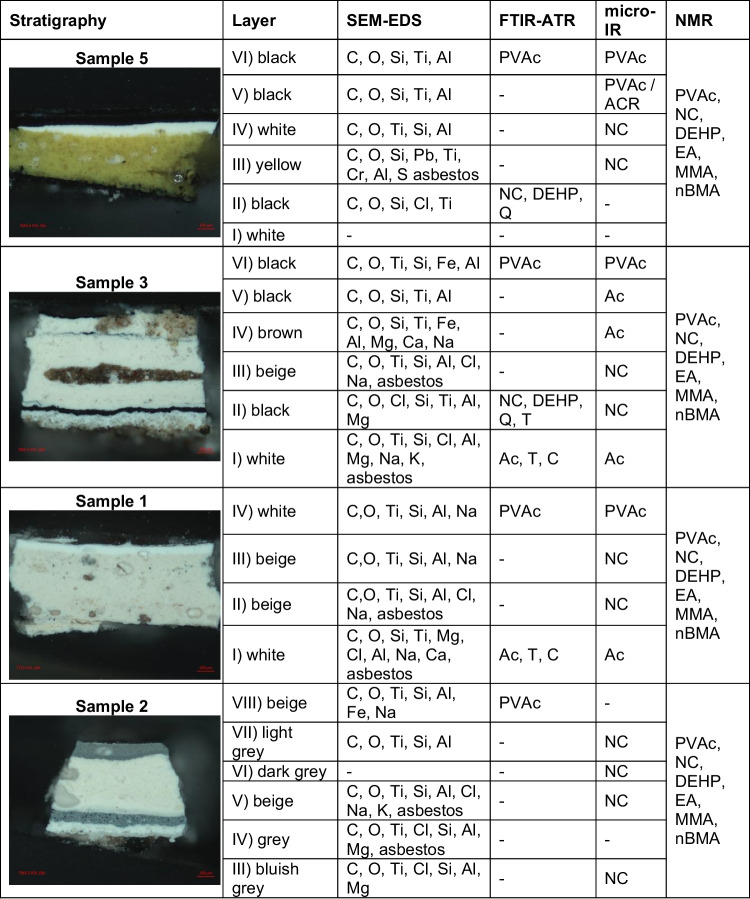

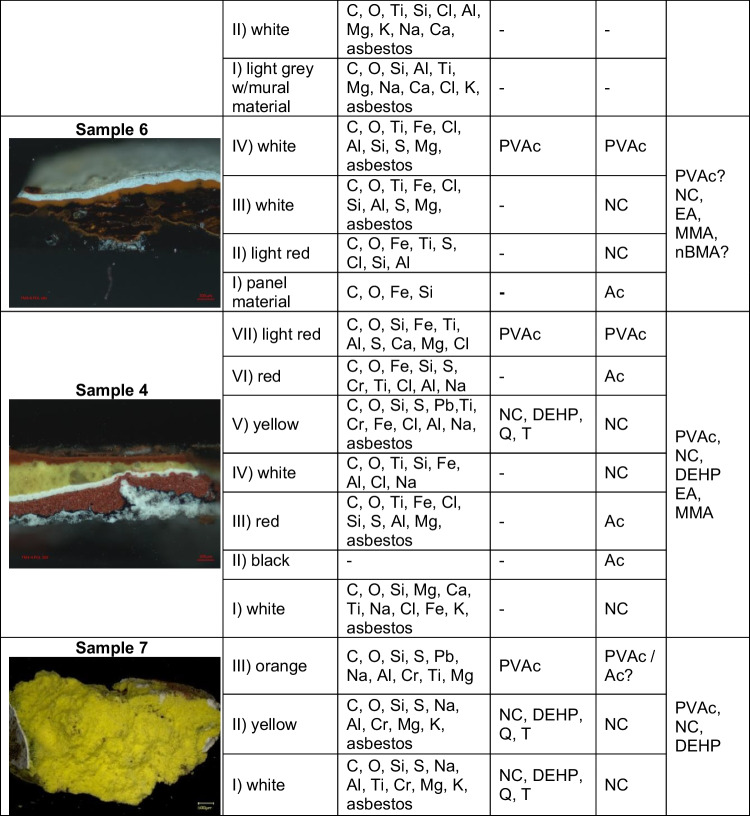
Summary of the composition identified in each pictorial layer through the analytical techniques

*PVAc* polyvinyl acetate, *NC* nitrocellulose, *DEHP* diethylhexyl phthalate, *Ac* acrylic resin, *T* talc, *Q* quartz, *C* CaCO_3_, *MMA* methyl methacrylate, *nBMA* n-butyl methacrylate, *EA* ethyl acrylate

Regarding the pigments, in *Untitled Mural 3*, commonly used compounds as titanium white, iron red oxide, and possibly carbon black were identified. Since S, Cr, and Pb were detected, it was proposed that the yellow pigment is chrome yellow.

As binders, nitrocellulose, different acrylic compositions, and PVAc were identified in all samples. PVAc was almost always present in the layers closer to the surface. This binder was not found in *Trazos de composición piramidal.* These unexpected findings contravene the historical records which indicate that Siqueiros predominantly used acrylic resins for outdoor murals. Since *Untitled Mural 3* had not previously undergone restoration, and no paint was added after Siqueiros intervention, the presence of PVAc polymer suggests the possibility that the artist may have selected *vinelita* or vinylic painting techniques [13], highlighting the need for further research and exploration into Siqueiros’ diverse artistic practices.

Moreover, the confirmation of nitrocellulose and the plasticizer DEHP in all the samples provides further evidence of Siqueiros’ experimentation with various binders. The data obtained from NMR analysis allows the distinction of different nitrocellulose monomers. These characterized two nitrocellulose monomers may indicate also the use of two distinct painting formulations, batches, or brands. In *Untitled Mural 3*, only DEHP plasticizer was detected, while in the *Trazos de composición piramidal*, there were several others.

In the previous study, nitrocellulose binders were identified although it was unclear whether if they were applied during a restoration process or if they were an artistic choice. In the actual study, the presence of nitrocellulose in many underlying layers of *Untitled Mural 3*, which has never been restored, confirms that it was used consciously by Siqueiros and it is not the result of a conservation process. This constant presence across multiple artworks serves to emphasize the continuous use of nitrocellulose in Siqueiros’ artistic repertoire since the 1930s decade, as well as its mixture with asbestos fillers in the ground layers. Siqueiros’s experimentation with various binders is evident, suggesting a lack of consistent application order.

The random combination of intermingled layers of different binders makes it difficult to predict the behavior of the painting as a system, especially because all these binders provide diverse ranges of flexibility and resistance to the stress, and environmental conditions.

## Conclusions

The analysis of *Untitled Mural 3* revealed significant findings regarding its chemical composition. Several binders were identified: PVAc, acrylics, and two monomers of nitrocellulose. These findings provide valuable insights into Siqueiros’ artistic technique, in a mural painted between 1964 and 1971, and demonstrate the extensive period of time during which the painter utilized nitrocellulose lacquers. It has been reported that he began using nitrocellulose around 1935, but the historical record registered that he used mostly acrylics in his later paintings. This study demonstrates that he used nitrocellulose until the 1970s.

Due to the complexity and apparent randomness of several layered materials used by Siqueiros, to have a precise characterization of all the organic and inorganic components of each layer it was necessary to recur to sampling. This research proposes a methodology to determine a reliable characterization with approximately 1 mg of sample. The integration of spectroscopic and microscopic results allowed the precise characterization of each layer.

This study highlights the importance of using rigorous analytical methods to extract as much information as possible from a limited amount of sample, ultimately contributing to a deeper understanding of artistic techniques and material choices.

## Supplementary Information

Below is the link to the electronic supplementary material.

Supplementary file1 (PDF 1863 KB)

## Footnotes

1 Rohm and Haas Reporter, Volume XXVI, Number 6 November December 1968 ‘‘Mexico lands of Murals” 4–8.

2 Biol. Sandra Joyce Ramírez Muñoz, Fumigation report, CENCROPAM-INBAL Archive, consulted march 5^th^, 2019. “The *Untitled Mural 3* was fumigated with *Citrik Restauración*® at 10% by nebulization.”
