# Supplementary material for: Challenges of analytical methods for the characterization of microsamples from David Alfaro Siqueiros mural painting
Source: Anal Bioanal Chem. 2024 Nov 13;417(1):143–54. doi: 10.1007/s00216-024-05633-x (PMC11695650; doi:10.1007/s00216-024-05633-x)
Supplement: Supplementary file 1 — Supplementary file1 (PDF 1863 KB) [file 216_2024_5633_MOESM1_ESM.pdf]

Challenges of analytical methods for the characterization of micro-samples from David Alfaro Siqueiros mural painting

Adrián Mejía-González[a§], Yareli Jáidar[b§], Pablo Aguilar-Rodríguez[a§], Sandra Zetina[b§], Nuria Esturau-Escofet[a*§]

[a] Instituto de Química, Universidad Nacional Autónoma de México, Mexico City, Mexico.

[b] Instituto de Investigaciones Estéticas, Universidad Nacional Autónoma de México, Mexico City, Mexico.

[*]Corresponding author: Nuria Esturau-Escofet, Instituto de Química, Universidad Nacional Autónoma de México, México. D.F., C.P. 04510, México. E-mail: nesturau@iquimica.unam.mx.

ORCID 0000-0002-0915-5346

[§] Members of the Laboratorio Nacional de Ciencias para la Investigación y la Conservación del Patrimonio Cultural (LANCIC, National Science Laboratory for Research and Conservation of Cultural Heritage)

Table S1 OM of the cross-sections, front and back of the samples. Assignation, general description and EDS results from the painting layers

Table S2 Elements identified by SEM-EDS. Stratigraphic analysis by area of the painting layers from the sample **5**

Fig. S1 a) SEM micrograph (BSE, 15.0 kV, 1500x) close-up of the red **III** painting layer of sample **2**. Elemental mappings (EDS, 15.0 kV, 1500x) of predominant elements: b) Overlapped elemental mappings, c) Si, d) Na, e) Mg, f) Ca, g) Ti, h) Al and i) K

Fig. S2 ATR-FTIR spectra of reference materials. a) PVAc resin, b), nitrocellulose lacquer, c) DEHP, d) EA-MMA dimers in a commercial paint, e) talc, f) $CaCO_3$, g) quartz and h) water. Characteristic bands are indicated

Fig. S3a micro-FTIR reflectance spectra from synthetic resins: a) acrylic inclusion resin b) frontal black **5-V** y **5-VI** layers and c) commercial PVAc resin sample

Fig. S3b micro-FTIR reflectance spectra from nitrocellulose: a) white **5-IV** layer and b) commercial nitrocellulose lacquer sample

Table S3 Samples stratigraphy and binders identified through micro-FTIR reflectance spectra

Fig. S4a 2D NMR spectra (700 MHz, $CDCl_3$, 300.0 K): HSQC (red-blue) and HMBC (green) of sample **5**. The structure and assignment of the signals from the PVAc resin are shown

Fig. S4b 2D NMR spectra (700 MHz, $CDCl_3$, 300.0 K): HSQC (red-blue) and HMBC (green) of sample **5**. The structure of DEHP plasticizer and assignment of the signals are shown

Fig. S4c ^{1}H NMR spectra (700 MHz, $CDCl_3$, 300.0 K) of microsamples. a) sample 5, b) sample 3, c) sample 1, d) sample 2, e) sample 6, f) sample 4 and g) sample 7. The signals of the components: 1) PVAc, 2) DEHP, 3) MMA, 4) n-BMA y 5) EA are indicated

Fig. S4d edited-HSQC spectrum (700 MHz, DMSO-d_6, 300.0 K) of a commercial nitrocellulose lacquer sample. The structure of TNC and 2,6-DNC monomers and assignment of the signals are shown

Table S1 OM of the cross-sections, front and back of the samples. Assignation, general description and EDS results from the painting layers

Stratigraphy	Layer / thickness (µm) / Description	Main elements identified by EDS
**Sample 5** 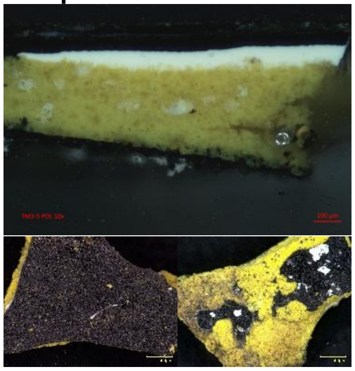	VI / 25 -30 / Homogeneous, compact and shiny black layers with some dispersed fillers	C, O, Si, Ti, Al
		C, O, Si, Ti, Al
	IV / 5 – 70/ Compact and homogeneous white layer	C, O, Ti, Si, Al
	III / 250 – 380 / Porous yellow layer with varying thickness, and heterogeneous, translucent and elongated crystalline material	C, O, Si, Pb, Ti, Cr, Al, S, asbestos
	II / 10 – 40 / Homogeneous, compact and shiny black layer, without fillers	C, O, Si, Cl, Ti
	I / – / Compact and homogeneous white layer	-
**Sample 3** 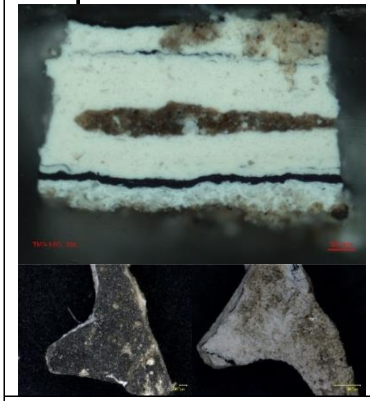	VI / 8 – 20 / Homogeneous, compact and shiny black layer, without fillers	C, O, Ti, Si, Fe, Al
	V / 1.5 – 16 / Thinnest homogeneous, compact black layer without fillers	C, O, Si, Ti, Al
	IV / 42 – 127 / Translucent resin with large white and brownish amorphous fillers	C, O, Si, Ti, Fe, Al, Mg, Ca, Na
	III / 531 – 549 / Thick beige layer with heterogeneous translucent fillers	C, O, Ti, Si, Al, Cl, Na, asbestos
	II / 19 – 34 / Homogeneous, compact and shiny black layer	C, O, Cl, Si, Ti, Al, Mg
	I / 56 – 112 / White porous layer with heterogeneous, translucent and elongated crystalline material over mural panel	C, O, Ti, Si, Cl, Al, Mg, Na, K, asbestos
**Sample 1** 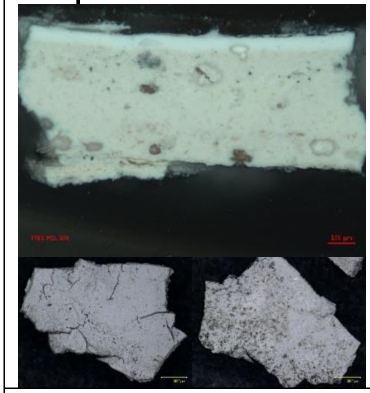	IV / 40 – 54 / Compact and homogeneous white layer	C, O, Ti, Si, Al, Na
	III / 384 – 410 / Thick beige layer with heterogeneous black and translucent fillers	C, O, Ti, Si, Al, Na
	II / 18 – 37 / Thin beige layer fragment with heterogeneous translucent fillers	C, O, Ti, Si, Al, Cl, Na, asbestos
	I / 18 – 36 / White layer with translucent and elongated crystalline material, probably asbestos fillers	C, O, Si, Ti, Mg, Cl, Al, Na, Ca, asbestos
**Sample 2** 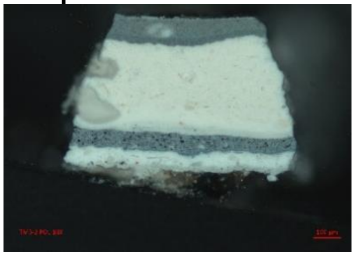	VIII / 4 – 9 / Thin homogeneous and compact beige layer	C, O, Ti, Si, Al, Fe, Na
	VII / 42 – 69 / Homogeneous and compact light gray layer with small black pigment	C, O, Ti, Si, Al
	VI / 16 – 29 / Homogeneous and compact dark gray layer with small black pigment	-
	V / 350 – 370 / Thick beige layer with heterogeneous translucent fillers	C, O, Ti, Si, Al, Cl, Na, K, asbestos
	IV / 40 – 64 / Compact light gray layer with black pigment, and translucent and elongated crystalline material	C, O, Ti, Cl, Si, Al, Mg, asbestos

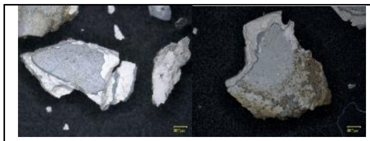	III / 5 – 20 / Homogeneous and compact bluish gray layer with small black pigment	C, O, Ti, Cl, Si, Al, Mg
	II / 31 – 61 / White porous layer with heterogeneous, translucent and elongated crystalline material	C, O, Ti, Si, Cl, Al, Mg, K, Na, Ca, asbestos
	I / 12 – 26 / Thin light grey porous layer with heterogeneous, translucent and elongated crystalline material over mural panel	C, O, Si, Al, Ti, Mg, Na, Ca, Cl, K, asbestos
**Sample 6** 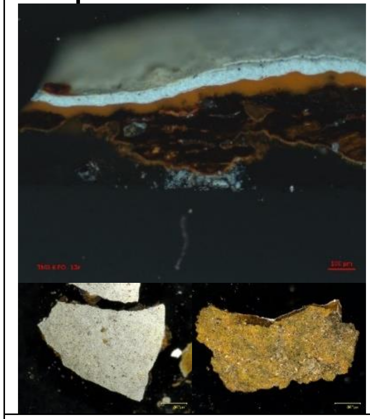	IV / 4 – 9 / White porous layer with translucent and elongated crystalline material, with some dispersed black pigment	C, O, Ti, Fe, Cl, Al, Si, S, Mg, asbestos
	III / 29 – 63 / White porous layer with translucent and elongated crystalline material, with some dispersed black pigment	C, O, Ti, Fe, Cl, Si, Al, S, Mg, asbestos
	II / 19 – 84 / Homogeneous and porous light red layer with dispersed white pigment	C, O, Fe, Ti, S, Cl, Si, Al
	I / 22 – 279 / Fragment of mural with brown color, appears to have oxidized material	C, O, Fe, Si
**Sample 4** 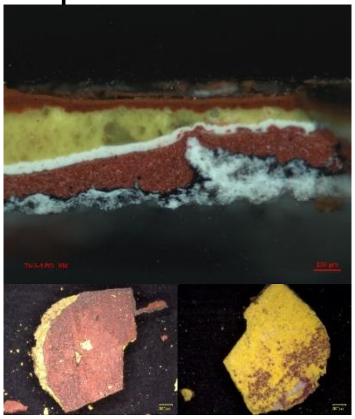	VII / 14 – 41 / Homogeneous and porous light red layer with translucent fillers	C, O, Si, Fe, Ti, Al, S, Ca, Mg, Cl
	VI / 11 – 70 / Homogeneous and porous red layer with irregular red and black fillers	C, O, Fe, Si, S, Cr, Ti, Cl, Al, Na
	V / 29 – 197 / Porous yellow layer with varying thickness, and heterogeneous, translucent and elongated crystalline material	C, O, Si, S, Ti, Cr, Fe, Cl, Al, Na, Pb, asbestos
	IV / 9 – 44 / Compact and homogeneous white layer with some red and black pigments	C, O, Ti, Si, Fe, Al, Cl, Na
	III / 21 – 156 / Thick red layer with elongated translucent and white fillers, also red and black pigments	C, O, Ti, Fe, Cl, Si, S, Al, Mg, asbestos
	II / 2 – 26 / Homogeneous and compact black layer intermingled with layer white	-
	I / 14 – 175 / White porous layer with heterogeneous, translucent and elongated crystalline material	C, O, Si, Mg, Ca, Ti, Na, Cl, Fe, K, asbestos
**Sample 7** 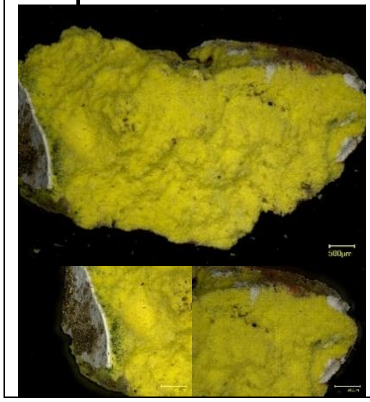	III / - / Homogeneous and porous orange layer	C, O, Si, S, Na, Al, Cr, Ti, Mg
	II / - / Porous yellow layer with varying thickness, and heterogeneous, translucent and elongated crystalline material	C, O, Si, S, Na, Al, Cr, Mg, K, asbestos
	I / - / White porous layer with heterogeneous, translucent and elongated crystalline material and brown material like sand	C, O, Si, S, Na, Al, Ti, Cr, Mg, K, asbestos

Table S2 Elements identified by SEM-EDS. Stratigraphic analysis by area of the painting layers from the sample **5**

Sample layer	Weight percentage (wt%)								
	C	O	Al	Si	S	Cl	Ti	Cr	Pb
**I) white**	-	-	-	-	-	-	-	-	-
**II) black**	65.0	25.0	0.2	5.0	-	4.3	0.3	-	-
**III) yellow**	58.3	32	0.3	6.8	0.1	-	0.3	0.3	1.6
**IV) white**	52.5	37.1	0.2	1.7	-	-	8.4	-	-
**V) black**	62.9	34.7	0.1	1.4	-	-	0.9		
**VI) black**	63.8	32.7	0.2	2.5	-	-	0.7		

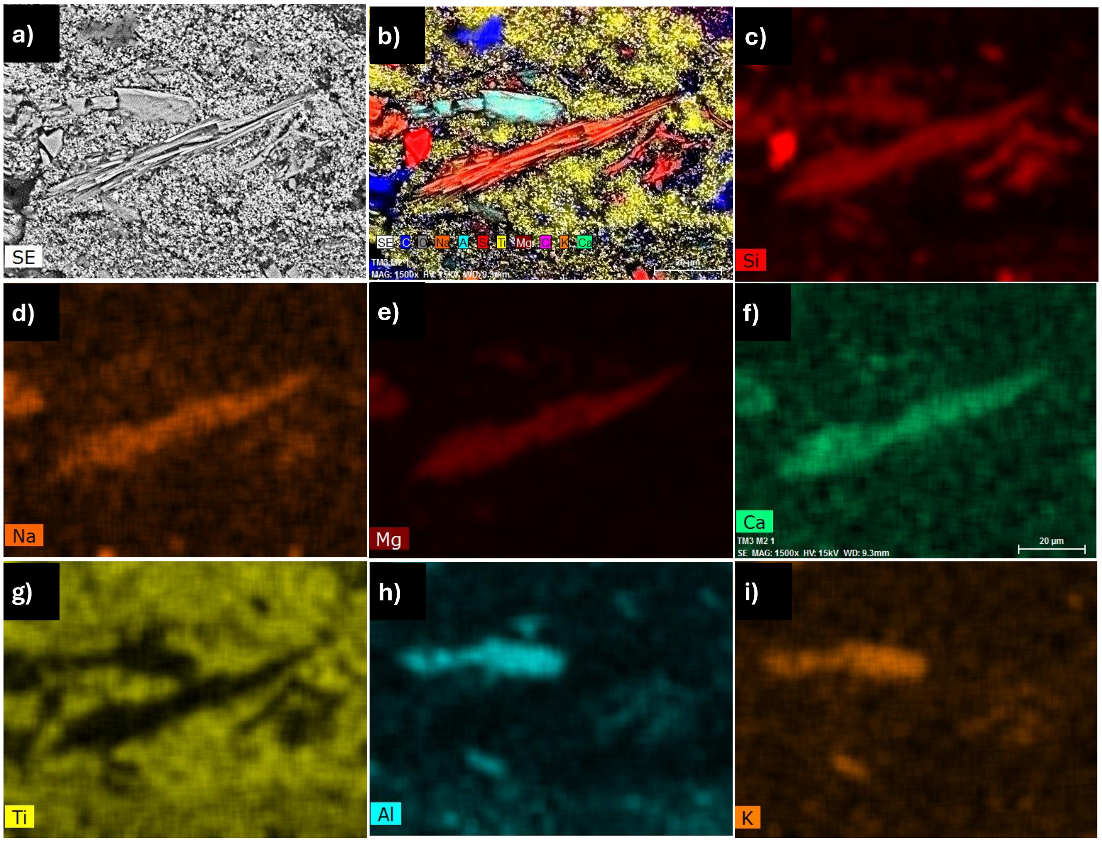

Fig. S1 a) SEM micrograph (BSE, 15.0 kV, 1500x) close-up of the red **III** painting layer of sample **2**. Elemental mappings (EDS, 15.0 kV, 1500x) of predominant elements: b) Overlapped elemental mappings, c) Si, d) Na, e) Mg, f) Ca, g) Ti, h) Al and i) K

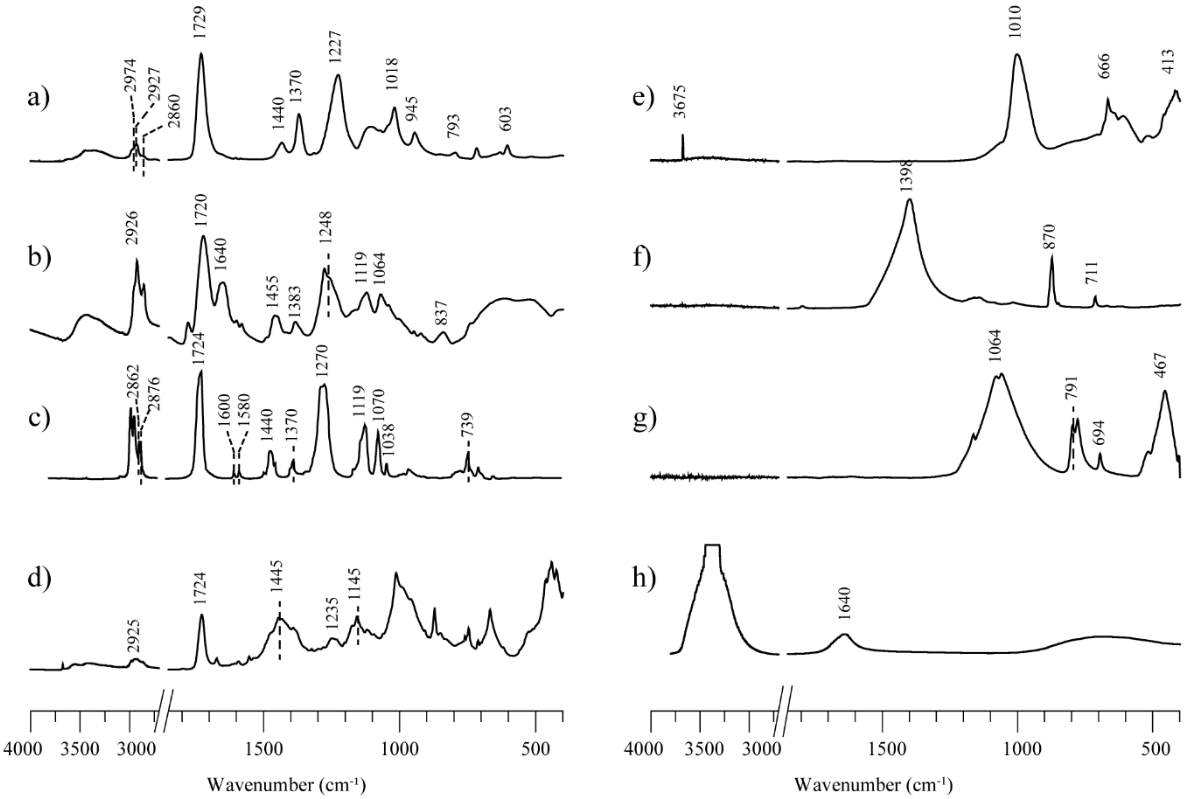

Fig. S2 ATR-FTIR spectra of reference materials. a) PVAc resin, b), nitrocellulose lacquer, c) DEHP, d) EA-MMA dimers in a commercial paint, e) talc, f) $CaCO_3$, g) quartz and h) water. Characteristic bands are indicated

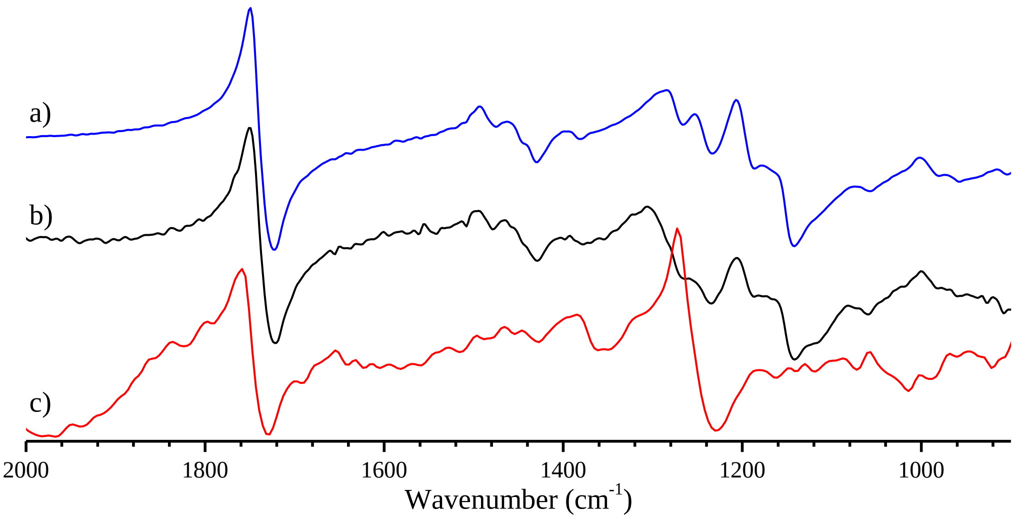

Fig. S3a micro-FTIR reflectance spectra from synthetic resins: a) acrylic inclusion resin b) frontal black **5-V** y **5-VI** layers and c) commercial PVAc resin sample

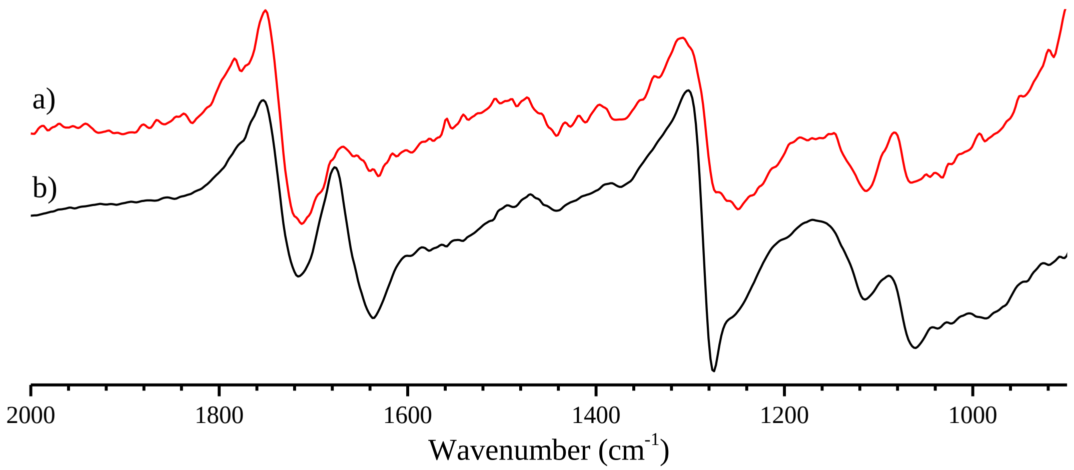

Fig. S3b micro-FTIR reflectance spectra from nitrocellulose: a) white **5-IV** layer and b) commercial nitrocellulose lacquer sample

Tab. S3 Samples stratigraphy and binders identified through micro-FTIR reflectance spectra

Stratigraphy	Layer	Identified binders
**Sample 5** 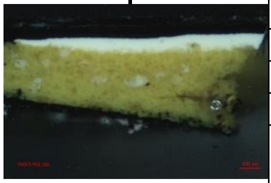	VI) black	PVAc
	V) black	PVAc
	IV) white	NC
	III) yellow	NC
	II) black	-
	I) white	-
**Sample 3** 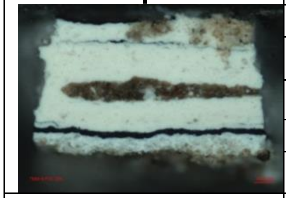	VI) black	PVAc
	V) black	Ac
	IV) brown	Ac
	III) beige	NC
	II) black	NC
	I) white	Ac
**Sample 1** 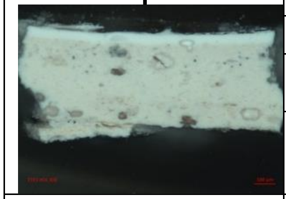	IV) white	PVAc
	III) beige	NC
	II) beige	NC
	I) white	Ac
**Sample 6** 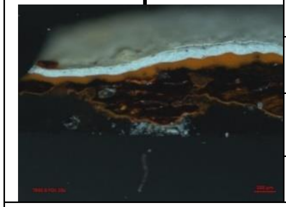	IV) white	PVAc
	III) white	NC
	II) light red	NC
	I) brown	Ac

Stratigraphy	Layer	Identified binders
**Sample 2** 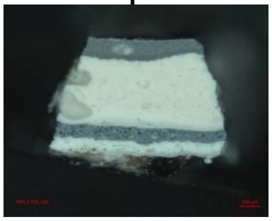	VIII) beige	-
	VII) light grey	NC
	VI) dark grey	NC
	V) beige	NC
	IV) grey	-
	III) bluish grey	NC
	II) white	-
	I) light grey	-
**Sample 4** 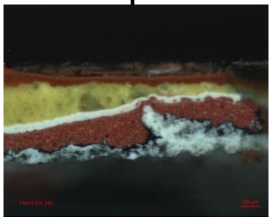	VII) light red	PVAc
	VI) red	Ac
	V) yellow	NC
	IV) white	NC
	III) red	Ac
	II) black	Ac
	I) white	NC
**Sample 7** 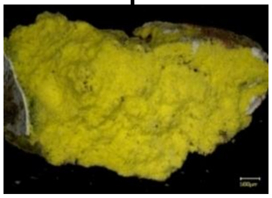	III) orange	PVAc
	II) yellow	NC
	I) white	NC

PVAc = polyvinyl acetate, NC = nitrocellulose, Ac = acrylic paint

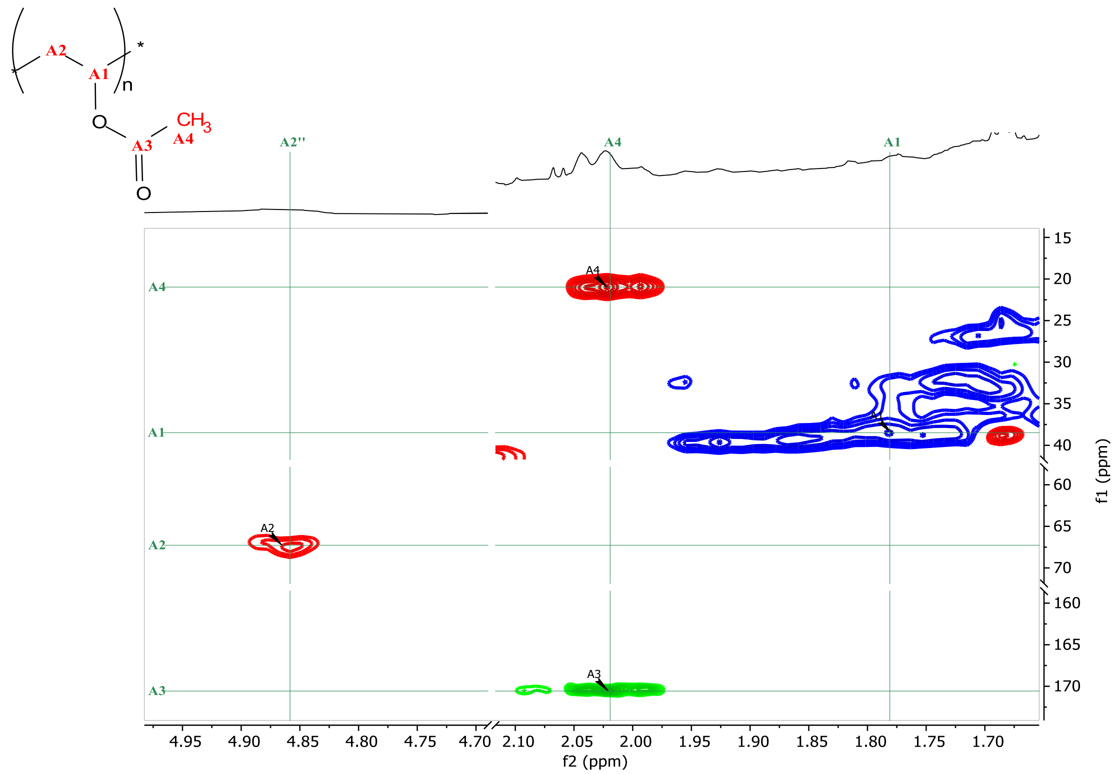

Fig. S4a 2D-NMR spectra (700 MHz, $CDCl_3$, 300.0 K): HSQC (red-blue) and HMBC (green) of sample **5**. The structure of PVAc resin and assignment of the signals are shown

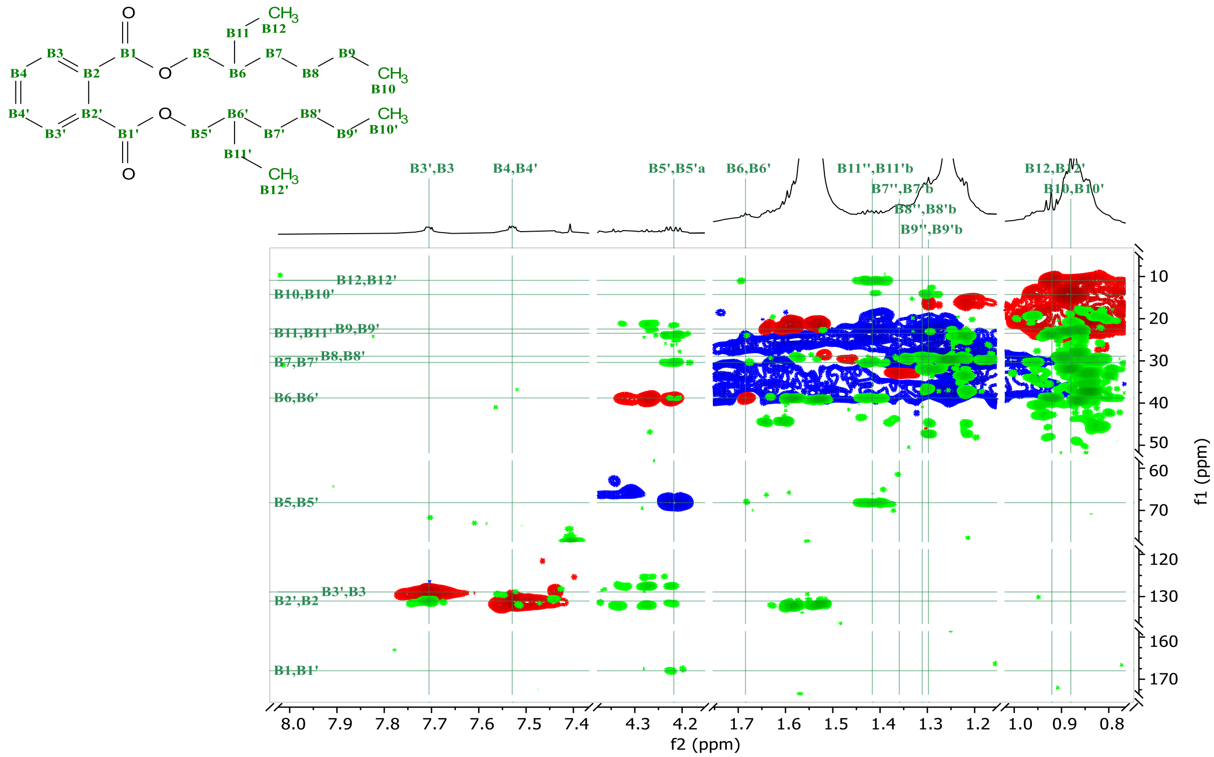

Fig. S4b 2D-NMR spectra (700 MHz, $CDCl_3$, 300.0 K): HSQC (red-blue) and HMBC (green) of sample **5**. The structure of DEHP plasticizer and assignment of the signals are shown

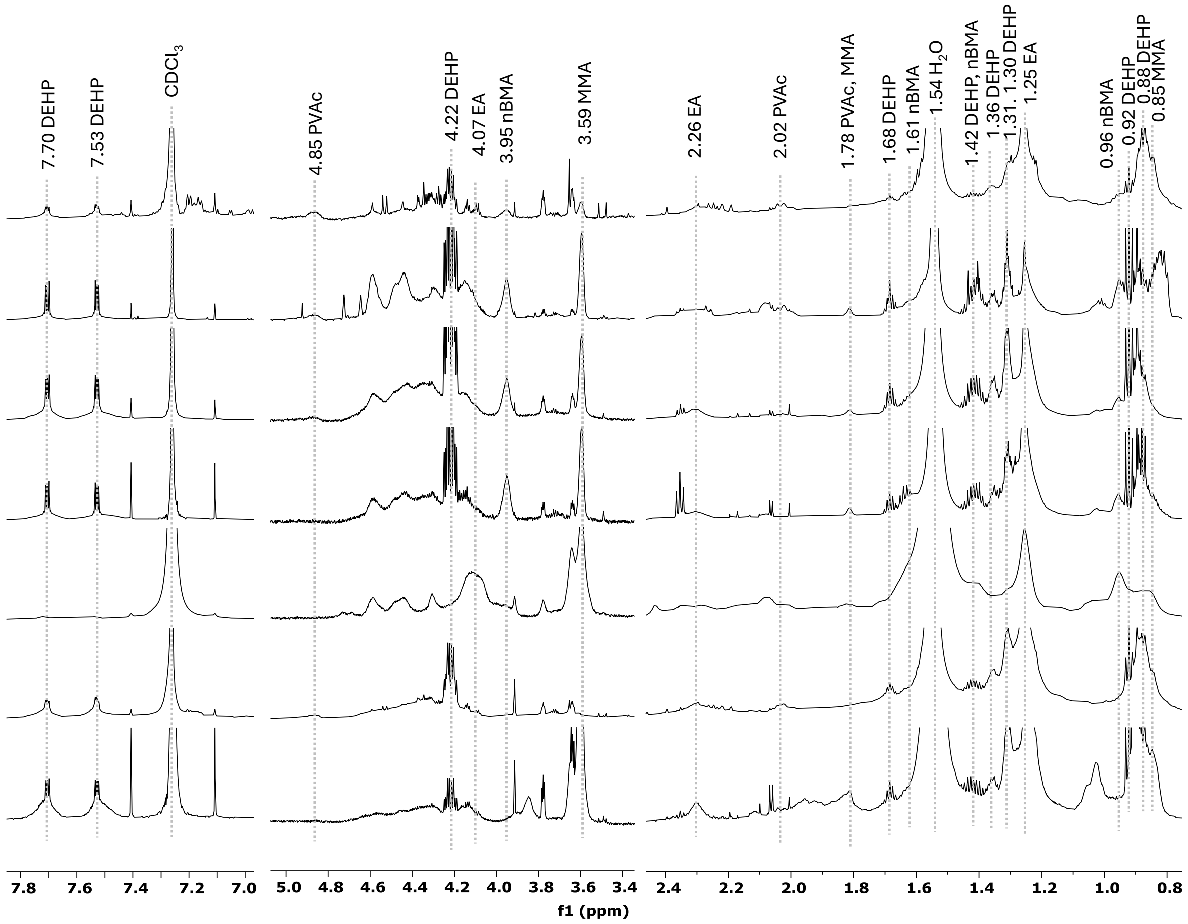

Fig. S4c ^{1}H-NMR spectra (700 MHz, $CDCl_3$, 300.0 K) of microsamples: a) **5**, b) **3**, c) **1**, d) **2**, e) **6**, f) **7** and g) **4**. The signals of the components: 1) PVAc, 2) DEHP, 3) MMA, 4) nBMA y 5) EA are indicated

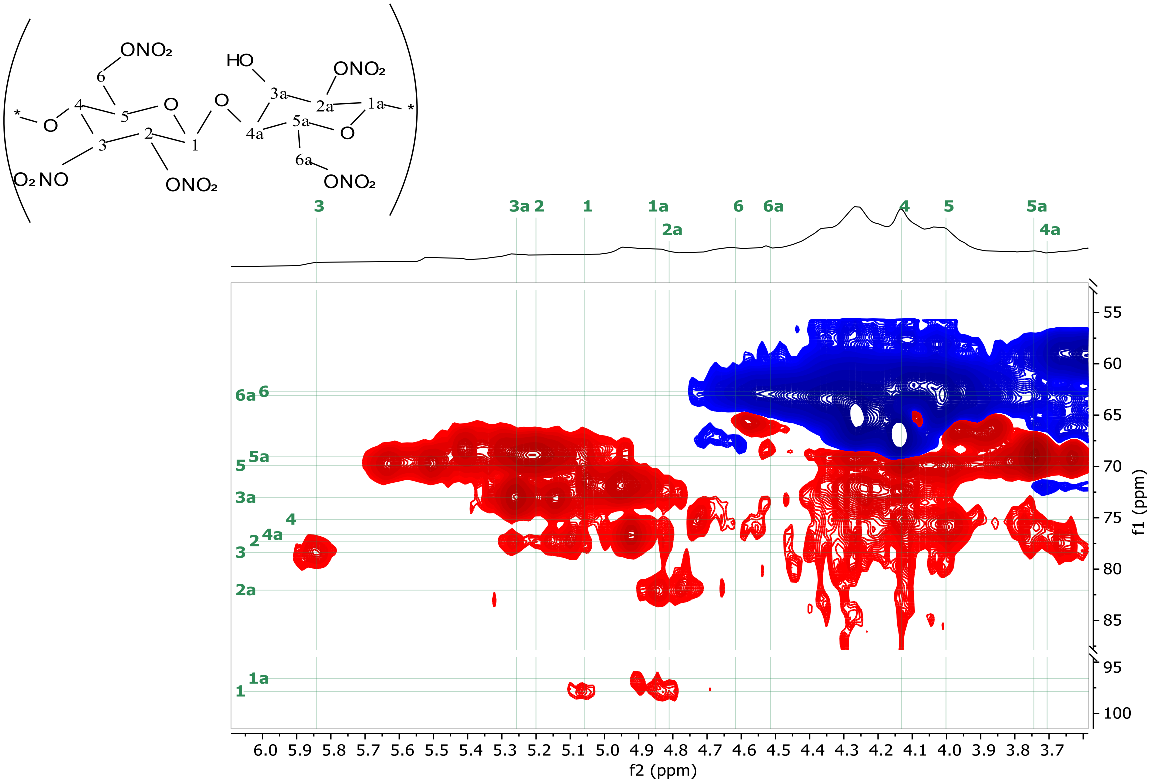

Fig. S4d edited-HSQC spectrum (700 MHz, DMSO-d_6, 300.0 K) of a commercial nitrocellulose lacquer sample. The structure of TNC and 2,6-DNC monomers and assignment of the signals are shown
